# Insulin-like Growth Factor II Prevents MPP+ and Glucocorticoid Mitochondrial-Oxidative and Neuronal Damage in Dopaminergic Neurons

**DOI:** 10.3390/antiox11010041

**Published:** 2021-12-24

**Authors:** Silvia Claros, Pablo Cabrera, Nadia Valverde, Silvana Y. Romero-Zerbo, Manuel Víctor López-González, Kirill Shumilov, Alicia Rivera, Jose Pavia, Elisa Martín-Montañez, María Garcia-Fernandez

**Affiliations:** 1 Department of Human Physiology, Faculty of Medicine, Biomedical Research Institute of Malaga, Malaga University, 29010 Malaga, Spain; silviacg@uma.es (S.C.); yaninaromero@uma.es (S.Y.R.-Z.); manuelvictor@uma.es (M.V.L.-G.); 2 Department of Pharmacology and Paediatrics, Faculty of Medicine, Biomedical Research Institute of Malaga, Malaga University, 29010 Malaga, Spain; pablo.cabrera.sspa@juntadeandalucia.es (P.C.); nadiavm@uma.es (N.V.); pavia@uma.es (J.P.); 3 Department of Cell Biology, Faculty of Science, Biomedical Research Institute of Malaga, Malaga University, 29010 Malaga, Spain; kirill@wustl.edu (K.S.); arivera@uma.es (A.R.); 4 School of Medicine, Washington University in St. Louis, St. Louis, MO 63101, USA

**Keywords:** insulin-like growth factor II, oxidative stress, mitochondria, neuroprotection, hormonal stress, Parkinson’s disease

## Abstract

Stress seems to contribute to Parkinson’s disease (PD) neuropathology, probably by dysregulation of the hypothalamic–pituitary–adrenal axis. Key factors in this pathophysiology are oxidative stress and mitochondrial dysfunction and neuronal glucocorticoid-induced toxicity. The insulin-like growth factor II (IGF-II), a pleiotropic hormone, has shown antioxidant and neuroprotective effects in some neurodegenerative disorders. Our aim was to examine the protective effect of IGF-II on a dopaminergic cellular combined model of PD and mild to moderate stress measuring oxidative stress parameters, mitochondrial and neuronal markers, and signalling pathways. IGF-II counteracts the mitochondrial-oxidative damage produced by the toxic synergistic effect of corticosterone and 1-methyl-4-phenylpyridinium, protecting dopaminergic neurons from death and neurodegeneration. IGF-II promotes PKC activation and nuclear factor (erythroid-derived 2)-like 2 antioxidant response in a glucocorticoid receptor-dependent pathway, preventing oxidative cell damage and maintaining mitochondrial function. Thus, IGF-II is a potential therapeutic tool for treatment and prevention of disease progression in PD patients suffering mild to moderate emotional stress.

## 1. Introduction

The neurodegenerative Parkinson’s disease (PD) affects 1–3% of the population aged over 65 [1]. A wide range of pathways and mechanisms are involved in its pathogenesis, such as oxidative stress, mitochondrial dysfunction, and inflammation [2,3], which ultimately produce a progressive loss of nigral dopamine neurons [3,4]. Dopamine depletion leads to the development of major motor symptoms that are key to diagnosis, although the disease comes with other non-motor symptoms, such as sleep disorders, cognitive impairment, and depression, which increase disability [2]. Repeated and persistent occurrence of emotional stress seems to promote the development of neurodegenerative diseases [5,6], and it has been suggested that a dysregulation of the hypothalamic–pituitary–adrenal axis (HPA) arises in PD [7]. This dysregulation may be involved in triggering, exacerbation, or progression of PD. Thus, the identification of new targets and the concomitant design of specific therapeutic approaches could slow the progression of the disease and/or improve symptomatology of PD patients [8].

Hypothalamic–pituitary–adrenal axis activation leads to the elevation in levels of glucocorticoid (GC), which performs important adaptive functions; however, the exposure of neurons to chronic stress often ends in maladaptation [9]. This long-term exposure to GC increases the possibility of neurodegenerative processes [10] and induces mitochondrial dysfunction [11]. Thus, the treatment of neuronal cell cultures from adult rats with corticosterone (CORT) induces oxidative damage, neurodegeneration, and cell death [12,13]. Stress has long been postulated to contribute to PD neuropathology, probably by increasing the susceptibility of dopamine neurons from midbrain to degeneration [14]. There exist strong indications of the role of GC through its interaction with glucocorticoid receptors (GRs) in the pathophysiology of PD, suggesting a deregulated HPA axis, which is known to affect GR activity [15]. Thus, compromised GR signalling is likely to contribute to neurodegeneration in PD [16]. The effect of mild to moderate hormonal stress seems to aggravate the pathology of PD, with a crucial role in mitochondrial-oxidative damage [17]. Therefore, excessive stress leading to chronically raised GC levels may aggravate PD pathology [18].

Insulin-like growth factor II (IGF-II) is a pleiotropic hormone with a widespread distribution in the central nervous system and numerous functions involving cellular homeostasis. IGF-II mediates its functions through the interactions with the specific receptor, the IGF-II/Mannose 6-Phosphate receptor (IGF-IIR) and, to a lesser extent, with insulin-like growth factor I receptor (IGF-IR) and insulin receptors (InRs). In adults, a high expression of IGF-IIR is found in a number of areas including the hippocampus, midbrain, and substantia nigra [19,20]. Although the specific functions of IGF-II are still poorly understood in adults, new evidence is emerging on the role of IGF-II as a key neuroprotective factor in pathological conditions, the study of IGF-II in neurodegenerative diseases being of increasing interest [21,22,23]. To date, only a small number of studies relate the neuroprotective mechanisms of IGF-II to its antioxidant function [12,13,23,24,25,26,27,28]. Thus, IGF-II has been shown to exhibit neuroprotective actions in Alzheimer’s disease, PD and ageing conditions [24,25,28], glucocorticoid-mediated stress situations [12,13], and neuropsychiatric disorders such as schizophrenia and autism [29,30,31].

Based on the above and given the solid association between PD and mild to moderate stress, the aim of this work was the study of the IGF-II neuroprotective effects against oxidative damage on a cellular combined model of PD and mild to moderate stress [17], based on corticosterone, an endocrine response marker to stress, and the dopaminergic neurotoxin 1-methyl-4-phenylpyridinium (MPP^+^). Our study shows that IGF-II administration prevents oxidative distress and maintains mitochondrial function in this in vitro combined model of PD. Thus, IGF-II may be a suitable therapeutic tool for the prevention and treatment of PD patients suffering mild to moderate emotional stress.

## 2. Materials and Methods

### 2.1. Cell Culture

The dopaminergic neuronal cell line SN4741 (RRID:CVCL_S466) derived from mouse substantia nigra [32] was grown to 70–80% confluence in culture flasks with D-MEM high-glucose containing 10% FBS, 1% penicillin/streptomycin, and 2 mM L-glutamine (Thermo Fisher Scientific, Waltham, MA, USA). Then, cells were detached with 0.25% trypsin and plated in 6-well plates at a concentration of 200,000 cells/well. For mitochondrial O_2_ consumption and immunocytochemistry, wells were pre-coated with 100 μg/mL of poly-d-lysine. Cells were treated with 200 μM MPP^+^, 0.5 μM CORT [17] (Merck/MilliporeSigma, Burlington, MA, USA), and 25 ng/mL IGF-II (provided by Lilly Laboratories, Madrid, Spain) for 2.5 or 6 h [17,24]. The treatments were applied in a modified Locke’s solution (137 mM NaCl, 5 mM CaCl_2_, 10 mM KCl, 25 mM glucose, 10 mM Hepes, pH: 7.4) supplemented with 1% penicillin/streptomycin and 2 mM l-glutamine. BMS-536924 1 μM (Tocris Bioscience, Bristol, UK) was used to inhibit the tyrosine kinase effect of IGF-IR and InRs. Mifepristone (MIFE) 10 μM (Merck/MilliporeSigma, USA), was used to inhibit the GC effect of the CORT.

### 2.2. Cell Viability

Quantification of the release of the intracellular enzyme lactate dehydrogenase was used to determine viability [33]. A commercial spectrophotometric assay kit (Randox Laboratories, Crumlin, UK) adapted to an ICubio AutoAnalyzer (ICubio Biomedical Technology, Shenzhen, China) was used to measure LDH levels in cell-free culture supernatants. The incubation time lasted 6 h.

### 2.3. Visualisation of Cell Morphology

In order to examine nuclear, cytoplasmic, and cell membrane changes after treatment, cells were fixed by adding 100% methanol, stained with 10% Giemsa solution (Merck/Millipore Sigma, USA) [34,35], and observed under an Olympus U-HGLPS model BX53F microscope. Bright images were then taken.

### 2.4. Determination of Oxidative Stress Parameters

#### 2.4.1. Mitochondrial Levels of ROS

Production of mitochondrial reactive oxygen species (ROS) was assessed by flow cytometry as the O_2_^•−^ production after 2.5 h of incubation using MitoSOX™ Red (Thermo Fisher Scientific, Waltham, MA, USA), following a previously published protocol [35,36]. Prior to incubation, cells were labelled with 2.5 µM MitoSOX in Locke’s solution, and then incubated at 37 °C for 30 min, washed, and analysed in an Accur^TM^ C6 flow cytometer (BD Biosciences, Franklin Lakes, NJ, USA). The FCS Express 5 software (De Novo Software, Pasadena, CA, USA) was used to analyse the events recorded (1 × 10^4^ Cells).

#### 2.4.2. Antioxidant Activity and Oxidative Cell Damage

Antioxidant enzyme activity of glutathione peroxidase (E.C.1.11.1.9) (GPX) was determined in homogenised cells at 37 °C using a commercial spectrophotometric assay kit (Randox Laboratories, Crumlin, UK) adapted to an ICubio AutoAnalyzer (ICubio Biomedical Technology, Shenzhen, China) [37]. One activity unit was expressed as the oxidation of NADPH (1 µmol) to NADP per minute at 37 °C. The fraction of the antioxidant pool (total antioxidant status, TAS) and the cell oxidative damage (as levels of lipid hydroperoxides, LOOH, and advanced-oxidation protein products, AOPP) were evaluated using spectrophotometric methods in cell homogenates [35,38]. After treatment (6 h), cells were suspended in 10 mM HEPES, 10 mM KCl (pH 7.4) with inhibitors of proteases (Thermo Fisher Scientific, USA) and phosphatases (Merck/MilliporeSigma, USA); then they were homogenised in the presence of 0.01% digitonin at 4 °C [12]. Bradford assay was used to determine protein concentrations.

### 2.5. Measurement of Mitochondrial Markers

#### 2.5.1. Mitochondrial Membrane Potential

The measurement of mitochondrial membrane potential (mΔΨ) was performed by using the lipophilic cationic probe 5,5,6′,6′-tetrachloro-1,1′,3,3′-tetraethyl benzimidazolcarbocyanine iodide (JC-1), following a previously published method [39]. Cells were exposed to 1 μg/mL JC-1 for 20 min at 37 °C, then rinsed twice, detached, and analysed in an Accuri^TM^ C6 flow cytometer (BD biosciences, USA) using FL1 and FL2 filters. The FCS Express 5 software (De Novo Software, Pasadena, CA, USA) was used to analyse the events recorded (1 × 10^4^ Cells). The potassium ionophore Valinomycin (1 µM) was used to completely deplete the mΔΨ and used as control. The lipophilic carbocyanine JC-1 exists as monomeric form and accumulates in the mitochondria; in the presence of high mΔΨ, JC-1 aggregates fluoresce in the orange/red channel (FL2-590 nm) after excitation at 488 nm. The collapse of mΔΨ produced a decrease in the number of JC-1 aggregates and an increase in the number of monomers that fluoresce in the green channel (FL1-525 nm). The mΔΨ was thus estimated from the red/green ratios as the FL2/FL1 ratio of JC1 staining.

#### 2.5.2. Mitochondrial Oxygen Consumption Rate

Mitochondrial O_2_ consumption rate (OCR) was monitored in real time using the Seahorse Bioscience XF24 analyser (Agilent Technologies, Santa Clara, CA, USA) [40,41]. Cells were seeded in 24-well plates (20,000 cells/well), washed with PBS, and then incubated with Agilent Seahorse XF Base Medium (without phenol red and bicarbonate) (590 µL) containing pyruvate (1 mM) and glucose (25 mM). The measurement of OCR was performed using the commercial Seahorse XF cell Mito Stress test kit (Agilent Technologies, USA). In brief, basal OCR was determined for 90 min before OCR recording after sequential injection of the mitochondrial toxins oligomycin, carbonyl cyanide-4-(trifluoromethoxy)phenylhydrazone (FCCP), and rotenone/antimycin. The addition of rotenone/antimycin A allowed obtaining values of non-mitochondrial respiration, which were used to correct all OCR values. Measurements were normalised according to protein concentration (Bradford).

### 2.6. Dopamine Marker and Neurodegeneration

The dopamine marker tyrosine hydroxylase (TH) was analysed by immunocytochemistry and confocal microscopy. Cells were fixed with methanol at −20 °C for 20 min. After rinsing in PBS, cells were incubated with rabbit anti-TH (1/5000; Merck/MilliporeSigma, USA) for 24 h at 4 °C. The cells were washed again with PBS and cells-bound primary antibody was then detected by incubating with goat anti-rabbit IgG conjugated with Alexafluor™ 488 (2 drops/mL; Thermo Fisher Scientific, USA) for 30 min at room temperature. Finally, the cells were mounted with Fluoromount™ (Merck/MilliporeSigma, USA). Microphotographs were acquired using a confocal microscope (LEICA SP5 II, Wetzlar, Germany) and processed using the software LAS AF Lite (Leica Microsystems AG, Germany). Neuronal degeneration was assessed by using the fluorescent Fluoro-Jade B™ (FJ) dye (Merck/MilliporeSigma, USA) following a previously published method [23,39]. Six hours after treatments, cells were seeded in 12-well plates (50,000 cells/well) and fixed with methanol as described above. Then, cells were covered with the dye (0.0004% FJ in 0.1% CH_3_COOH) and gently shaken in the dark at room temperature (30 min). The dye was then removed, and the fluorescence intensity measured using an FL600 (Bio Tek Instruments, USA) bottom-read mode fluorescence microplate reader (excitation/emission filters: 485/20 and 530/25, respectively).

### 2.7. Intracellular Signalling Pathways

Nuclear factor (erythroid-derived 2)-like 2 (Nrf2) and GR expressions were analysed by immunocytochemistry as described above. Dilutions of primary antibodies: anti-Nrf2 (1/1000; Santa Cruz Biotechnology, Santa Cruz, CA, USA); anti-GR (1/250; Abcam Cambridge, UK). Fluorescent secondary antibodies used: Alexafluor™ 488 and Alexafluor™ 568 (Thermo Fisher Scientific, USA). Phosphorylated protein kinase C alpha/beta (pPKCα/β) expression was determined by electrophoresis of cell lysates and Western blot using a semi-dry transfer system (Trans-Blot Turbo Transfer System, Bio-Rad Laboratories, Hercules, CA, USA). After blocking for 1 h in TBS-Tween containing 5% skimmed milk the membranes were incubated overnight at 4 °C with anti-pPKCα/β and anti-β actin (1/1000; Cell Signaling Technology, USA). Detection of proteins was performed using horseradish-peroxidase-conjugated secondary antibody (1/1500; Bio-Rad Laboratories, USA), an enhanced chemiluminescence reagent (SuperSignal™ West Pico PLUS, Thermo Fisher, USA). Quantification was carried out using Image Lab™ Software (Bio-Rad Laboratories, USA).

### 2.8. Statistical Analysis

One-way ANOVA was used to determine statistical differences. The multiple comparison post hoc Tukey’s test was used for all pairwise comparisons. Statistical significance was set at *p* < 0.05.

## 3. Results

### 3.1. Cell Viability and Morphology

**Figure 1:**
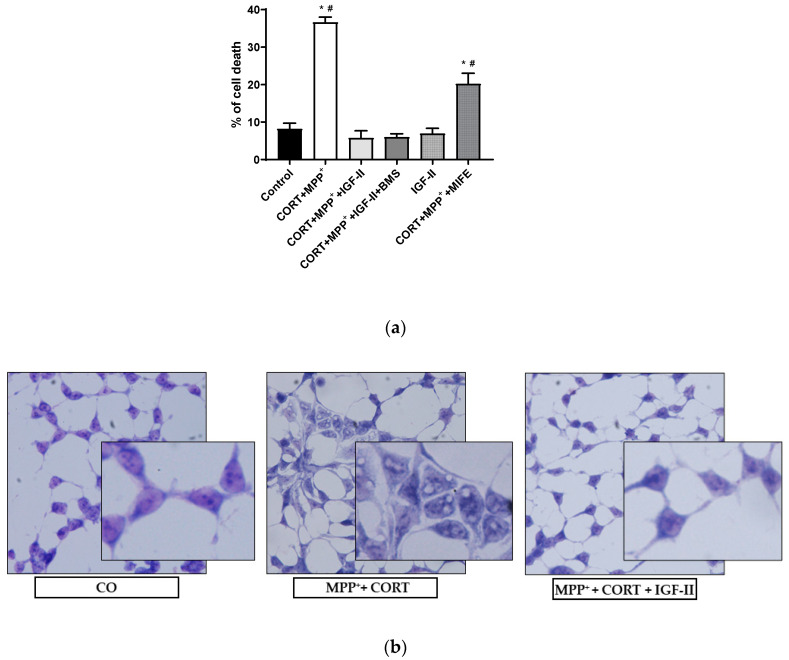
Cell death and morphology of SN4741 neuronal cells after 6 h of incubation with the combination of corticosterone and MPP^+^ (CORT + MPP^+^) in the presence or absence of IGF-II. (**a**) Cytotoxicity, measured by quantifying LDH release and expressed as % of control. BMS is used to define the specific receptor involved in the IGF-II effect; MIFE is used to inhibit the glucocorticoid effect of the CORT. (**b**) Giemsa staining of control cells; CORT + MPP^+^-treated cells; CORT + MPP^+^-treated cells in presence of IGF-II. Representative images (20X). Data from four independent experiments were combined and presented as mean ± SEM. * *p* < 0.05 compared to control cells; ^#^ *p* < 0.05 compared to all other groups.

Figure 1a shows the analysis of LDH released after incubation with the two drugs MPP^+^ and CORT. We found a dramatic 4.5-fold increase in LDH levels compared to those in controls (*p* < 0.05); co-incubation with IGF-II and both drugs prevented the release of LDH (*p* < 0.05), whereas incubation in the presence of BMS did not modify the effect of IGF-II. Cells solely in the presence of IGF-II showed similar LDH levels to those found in control, and co-incubation with MIFE and both drugs partially decreased the cell death found with the administration of both drugs (2.5-fold increase of LDH versus control cells, *p* < 0.05). Regarding the study of cell morphology, the analysis of the Giemsa-stained images (Figure 1b) showed strong morphological changes in cells exposed to CORT and MPP^+^ incubation compared to control, with clear heterogeneity in cell shape and swelling, and with shrunken condensed pyknotic nuclei. After the administration of IGF-II in combination with both drugs, cells showed a morphology similar to that found in the control cells.

### 3.2. Antioxidant Activity and Oxidative Cell Damage

**Figure 2:**
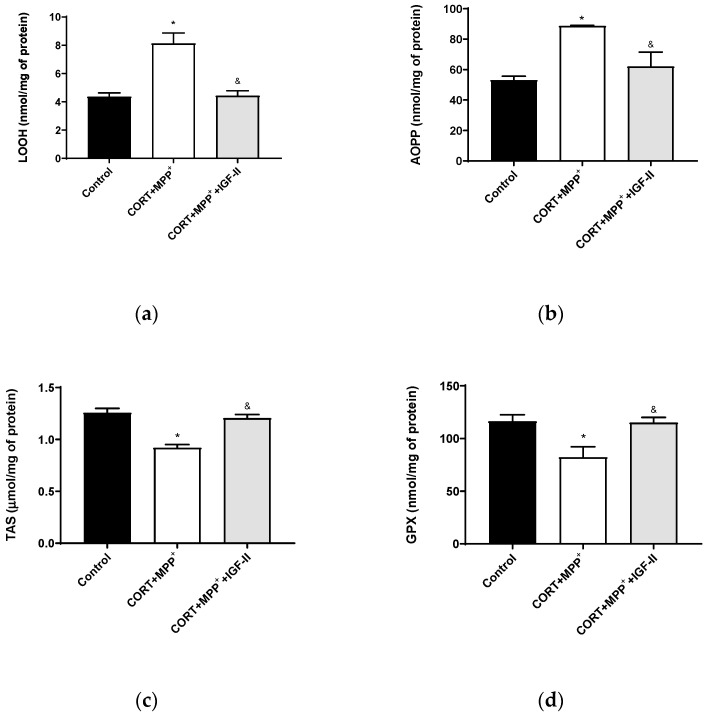
Oxidative chemistry biomarkers and antioxidant enzyme activity in SN4741 neuronal cells after 6 h of incubation with the combination of corticosterone and MPP^+^ (CORT + MPP^+^) in the presence or absence of IGF-II. (**a**) Levels of lipid hydroperoxides, LOOH; (**b**) levels of advanced-oxidation protein products, AOPP; (**c**) levels of total antioxidant status, TAS; (**d**) activity of glutathione peroxidase, GPX. Data from four to six independent experiments were combined and presented as mean ± SEM. * *p* < 0.05 compared to control cells; ^&^ *p* < 0.05 compared to CORT + MPP^+^ co-incubated cells.

In order to assess the oxidative cell damage, LOOH and AOPP were studied. LOOH (Figure 2a) and AOPP (Figure 2b) levels were dramatically increased in neurons treated with the combination of CORT+MPP^+^ (85 and 67%, respectively) compared to the control cells (*p* < 0.05). The inclusion of IGF-II in the treatment led LOOH and AOPP levels to values close to controls (*p* < 0.05). The TAS, i.e., the fraction of the antioxidant pool available for further anti-ROS activity, was significantly lower in cells incubated with the combination of CORT and MPP^+^ (27.7%, *p* < 0.05) compared to that in control cells, whereas their co-incubation with the drugs and IGF-II alleviated the reduction in TAS (*p* < 0.05) (Figure 2c). Incubation with both drugs also decreased the GPX activity compared to control (29.3%, *p* < 0.05) and again, co-treatment with IGF-II completely prevented the decrease in the enzyme activity (*p* < 0.05) (Figure 2d).

### 3.3. ROS Production and Measurement of Mitochondrial Function

Mitochondria are the main sources of pathological and physiological cellular free radical production, leading them to modify their function. In order to evaluate mitochondrial free radical production and mitochondrial function, O_2_^•−^ production, mΔΨ, and OCR were studied in the present model.

#### 3.3.1. Mitochondrial ROS Production and Mitochondrial Membrane Potential

**Figure 3:**
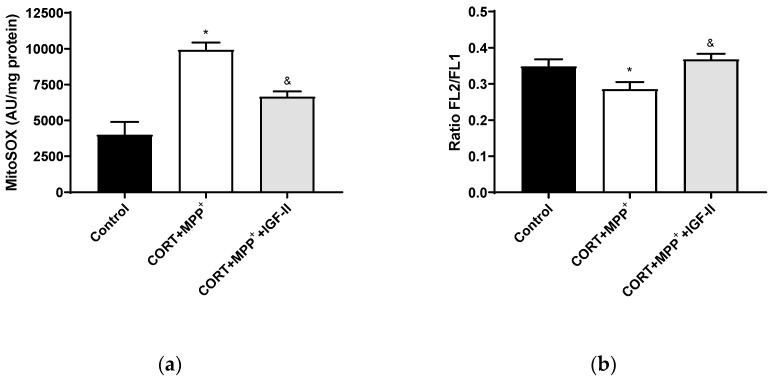
Mitochondrial markers in SN4741 neuronal cells after 2.5 h of incubation with the combination of corticosterone and MPP^+^ (CORT + MPP^+^) in the presence or absence of IGF-II. (**a**) Mitochondrial levels of ROS; (**b**) cytofluorometric analysis of mitochondrial membrane potential (mΔΨ). Data were combined from three to four independent experiments and presented as mean ± SEM. * *p* < 0.05 compared to control cells; ^&^ *p* < 0.05 compared with CORT + MPP^+^ co-incubated cells.

Treatment with CORT and MPP^+^ produced a dramatic 2.5-fold increase in MitoSOX™ Red fluorescence levels compared to those found in the control cells (*p* < 0.05), whereas IGF-II co-incubation took CORT+MPP^+^ free radical induction to values close to the control levels (*p* < 0.05, Figure 3a). Functionally, as observed in Figure 3b, incubation of cells with both drugs produced decreases (17.1% *p* < 0.05) in mΔΨ versus control cells. It was observed that the inclusion of IGF-II in the treatment maintained mΔΨ (*p* < 0.05).

#### 3.3.2. Mitochondrial Oxygen Consumption

**Figure 4:**
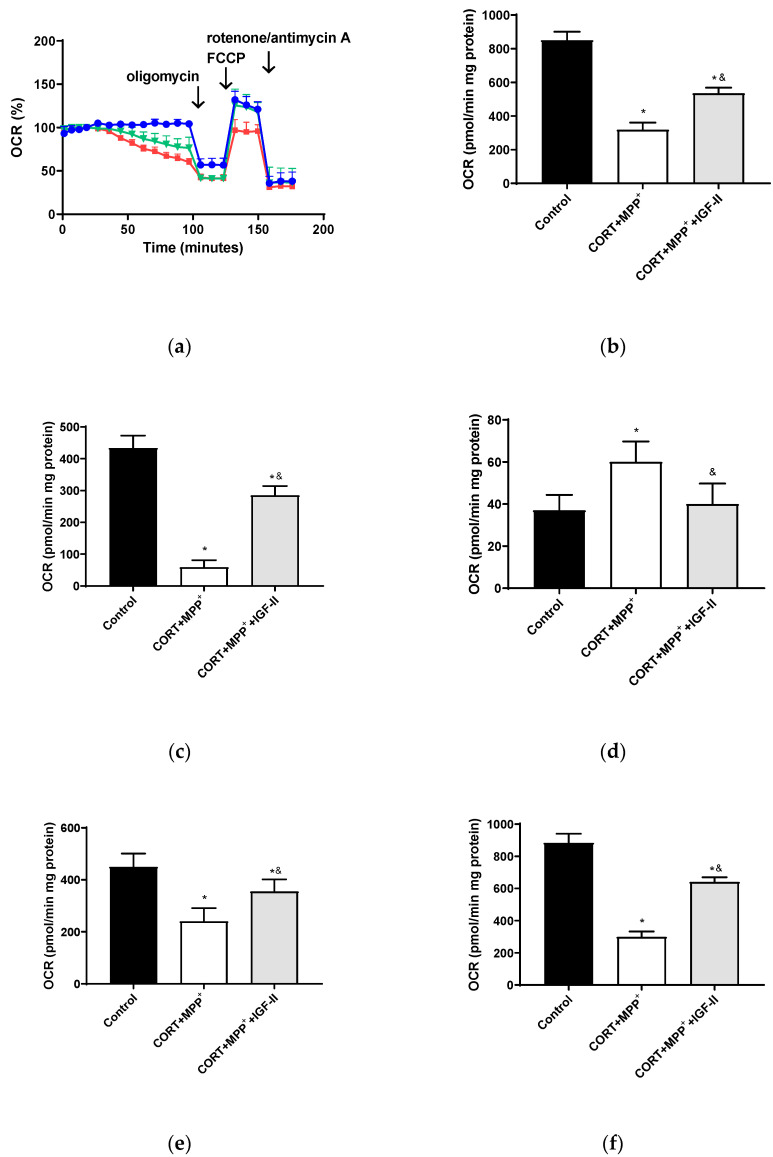
Study of the mitochondrial oxygen consumption rate (OCR) in SN4741 neuronal cells after 90 min of incubation with the combination of corticosterone and MPP^+^ (CORT + MPP^+^) in the presence or absence of IGF-II. (**a**) Representative experiment of the measurement of key parameters using the Mito Stress test kit. Blue line: control; green line: presence of IGF-II; red line: combination of the two drugs (CORT + MPP^+^); (**b**) basal consumption after 90 min of incubation; (**c**) ATP production; (**d**) proton leak; (**e**) spare respiratory capacity; (**f**) maximal respiration. Data from six to eight independent experiments were combined and presented as mean ± SEM. * *p* < 0.05 compared to control cells; ^&^ *p* < 0.05 compared to CORT + MPP^+^ co-incubated cells.

The bioenergetic assessment is shown in Figure 4. Thus, the administration of both drugs together produced a clear decrease in OCR of 62.5% after 2.5 h of incubation versus control cells (*p* < 0.05, Figure 4b), with this decrease being partially counteracted (37.1%) by co-incubation with IGF-II (*p* < 0.05). Regarding O_2_ consumption due to mitochondrial ATP synthesis and that related to SCR and maximal respiration (Figure 4c,e,f), large decreases were observed (86.4, 46.7, and 66.2%, respectively, *p* < 0.05). Again, IGF-II co-incubation alleviated these decreases (34.3, 21.1, and 27.6%, *p* < 0.05). Significant differences (62.2%) were also found in the remaining basal respiration, not linked to ATP production, after treatment with CORT+MPP^+^ compared to controls (*p* < 0.05, Figure 4d); the co-treatment with IGF-II and both drugs prevented the increase in OCR (*p* < 0.05).

### 3.4. Dopamine Markers and Neurodegeneration

The cell culture used in this PD model was a dopaminergic neuronal cell line derived from substantia nigra. In order to evaluate the neuronal integrity, specific dopamine and neurodegenerative markers were studied.

#### 3.4.1. Tyrosine Hydroxylase Expression

**Figure 5:**
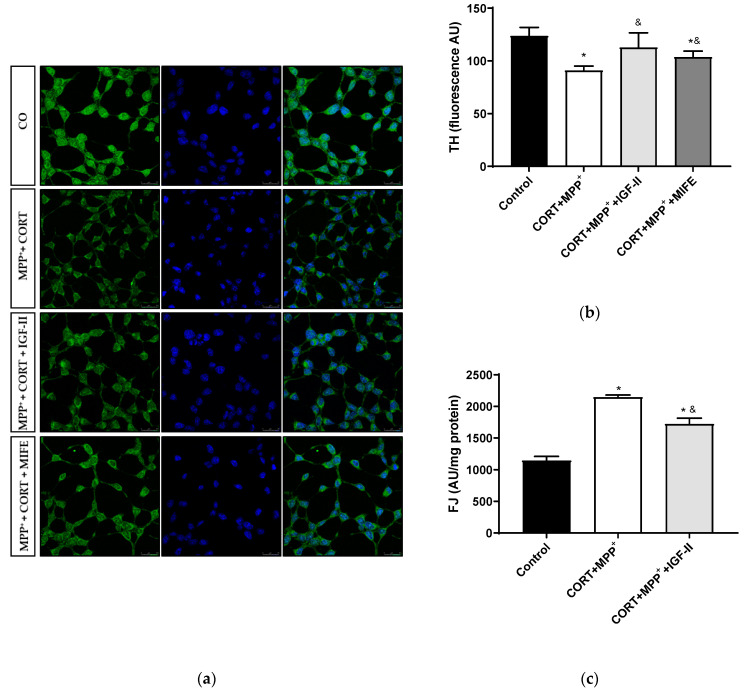
Study of dopaminergic marker and neurodegeneration in SN4741 neuronal cells after incubation with the combination of corticosterone and MPP^+^ (CORT + MPP^+^) in the presence or absence of IGF-II. (**a**) Representative immunocytochemistry stain for DAPI and TH; (**b**) Quantification of TH immunofluorescence; (**c**) Neurodegeneration evaluated as FJ fluorescence intensity. Representative images (63X). CO: control cells. Data from three independent experiments were combined and presented as mean ± SEM. * *p* < 0.05 compared to control cells; ^&^ *p* < 0.05 compared to CORT + MPP^+^ co-incubated cells.

The analysis of this dopamine marker is shown in Figure 5. Treatment with CORT and MPP^+^ led to a decrease of 24.7% in TH levels compared to those found in the control cells (*p* < 0.05), whereas IGF-II co-incubation took TH expression to values close to the control levels (*p* < 0.05, Figure 5a,b). The co-treatment with MIFE and both drugs partially prevented the decrease (13.9%, *p* < 0.05).

#### 3.4.2. Neurodegeneration

We examined cell degeneration using the specific stain FJ (Figure 5c). In these dopaminergic cells, a clear increase (87.3%) in the FJ intensity of the neurons treated with CORT and MPP^+^ was detected compared to the control (*p* < 0.05). This increase in FJ intensity produced by the co-treatment with these drugs was partially counteracted (50.2%) by co-incubation with IGF-II (*p* < 0.05).

### 3.5. Intracellular Signalling Pathways

**Figure 6:**
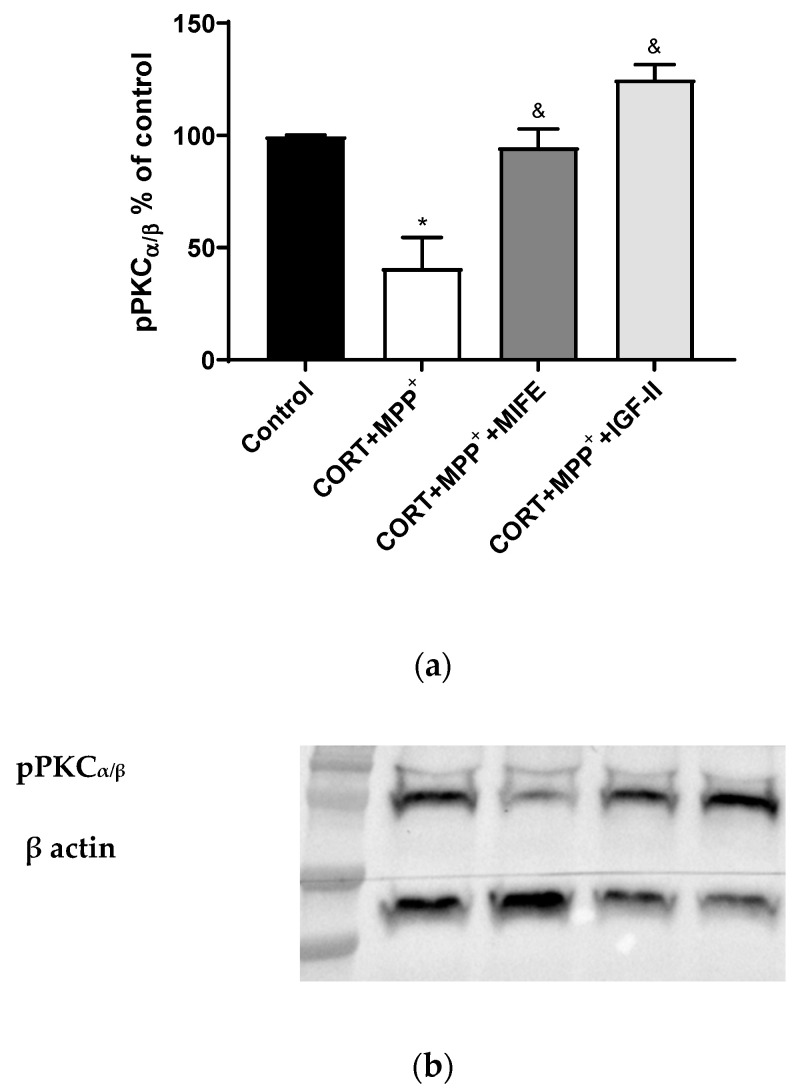
Study of PKCα expression in SN4741 neuronal cells after 2.5 h of incubation with the combination of corticosterone and MPP^+^ (CORT + MPP^+^) in the presence or absence of IGF-II. (**a**) Quantification after normalising with β actin; (**b**) Representative Western blot. Data from two independent experiments were combined and presented as mean ± SEM; each situation in each experiment carried out included three independent experiments with three samples in each one; * *p* < 0.05 compared to control cells; ^&^ *p* < 0.05 compared to CORT + MPP^+^ co-incubated cells.

It is known that PKC is modulated by GC, ROS, and IGF-II [42,43,44]. The administration of both drugs together produced a clear decrease in pPKCα/β expression of 58.8% after 2.5 h of incubation versus control cells (*p* < 0.05, Figure 6), whereas the co-treatment with MIFE and both drugs or the inclusion of IGF-II in the treatment led PKCα/β expression to values close to those of the control cells (*p* < 0.05).

**Figure 7:**
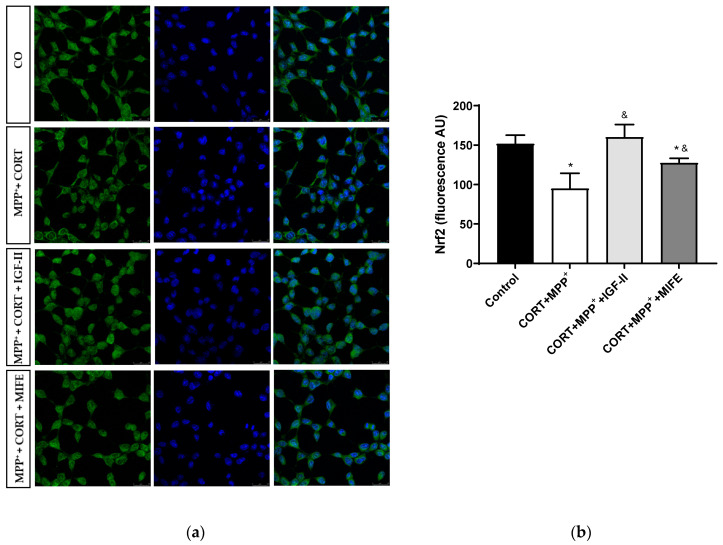
Study of Nrf2 expression in SN4741 neuronal cells after 2.5 h of incubation with the combination of corticosterone and MPP^+^ (CORT + MPP^+^) in the presence or absence of IGF-II. (**a**) Representative immunocytochemistry stain for DAPI and Nrf2 (63X). CO: control cells; (**b**) Quantification of Nrf2 immunofluorescence. Fluorescent intensity was estimated by number of cells per field. Data from three independent experiments were combined and presented as mean ± SEM. * *p* < 0.05 compared to control cells; ^&^ *p* < 0.05 compared to CORT + MPP^+^ co-incubated cells.

Nrf2 is a transcription factor functioning as a main regulator in controlling the endogenous antioxidant responses against oxidative distress. The assessment of its expression is shown in Figure 7 and Supplementary Materials Figure S1. No differences were observed in total expression between conditions; however, the administration of both drugs together produced a clear decrease in nuclear Nrf2 expression of 62.6% after 2.5 h of incubation versus control cells (*p* < 0.05), whereas the co-treatment with MIFE and both drugs partially prevented the decrease (28.3%, *p* < 0.05). The inclusion of IGF-II in the treatment took Nrf2 expression to values close to those of the control cells (*p* < 0.05).

**Figure 8:**
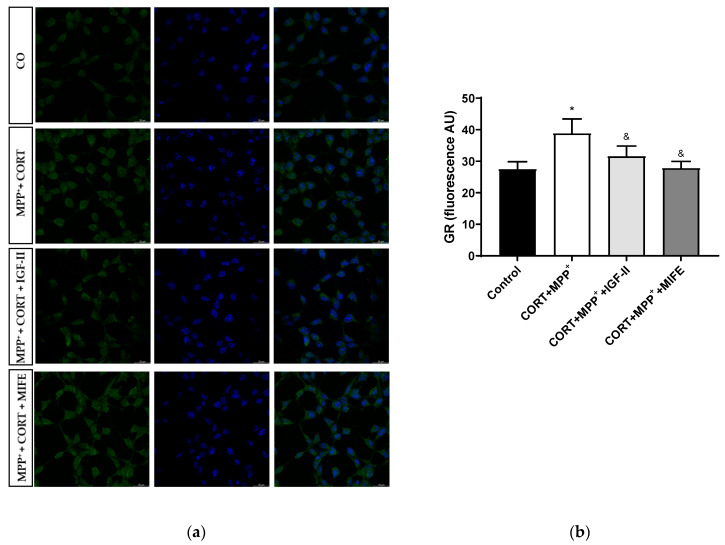
Study of GR expression in SN4741 neuronal cells after 2.5 h of incubation with the combination of corticosterone and MPP^+^ (CORT + MPP^+^) in the presence or absence of IGF-II. (**a**) Representative immunocytochemistry stain for DAPI and GR (63X). CO: control cells; (**b**) Quantification of GR immunofluorescence. Fluorescent intensity was estimated by number of cells per field. Data from three independent experiments were combined and presented as mean ± SEM. * *p* < 0.05 compared to control cells; ^&^ *p* < 0.05 compared to CORT + MPP^+^ co-incubated cells.

The nuclear GR expression could be considered a possible regulator of stress-related gene expression patterns [45]. Treatment with CORT and MPP^+^ produced an increase in nuclear GR expression compared to what was found in the control cells (41.4%, *p* < 0.05), whereas IGF-II co-incubation led CORT+MPP^+^ nuclear GR expression induction to values close to the control levels (*p* < 0.05); in the same way, the values found after co-incubation of both drugs with MIFE were close to the control (*p* < 0.05, Figure 8 and Supplementary Figure S1), inhibiting the nuclear translocation of the GR. No differences were observed in total expression between conditions.

## 4. Discussion

Results of the present work clearly demonstrated that CORT enhances dopaminergic cell damage induced by neurotoxin MPP^+^ [17], suggesting a connection between PD and mild to moderate stress. These results agreed with previous clinical reports finding that chronically GC levels in PD patients may impact PD pathology by aggravation of disease progression [14,46,47]. Additionally, in combined experimental models of drug-induced PD and mild to moderate emotional stress, the stressors were also shown to worsen the potential noxious effects of the toxins [17,48,49,50]. Together, all these findings pointed to GCs as risk factors for PD progression.

Parkinson disease and conditions of emotional stress are strongly related to increases in oxidative distress and mitochondrial damage, contributing to enlarged neurodegeneration and/or death [18,39,51,52,53]. The protective effect of IGF-II found in the cellular oxidative stress model induced by the CORT [12,13] and the PD models [24,27], which is mediated through its specific IGF-IIR, suggests a potential decrease in the mitochondrial-oxidative damage observed in this combined PD experimental model after IGF-II administration, leading to protecting dopaminergic neurons from cell death. Accordingly, the co-incubation with IGF-II and both drugs prevented the release of LDH, whereas the incubation in the presence of an inhibitor of IGF-IR and RIns (BMS) used to define the receptor involved in the IGF-II effect, did not modify the effect of IGF-II.

Parkinson’s disease and emotional stress are strongly related to ROS production. Thus, treatment with CORT and MPP^+^ produced a dramatic 2.5-fold increase in free radical production, decreasing the fraction of the antioxidant pool and also the activity of GPX (an enzyme that neutralised LOOH using GSH and NADH [54]), probably as a consequence of the increase in LOOH. Moreover, the observed decrease in GPX could be exacerbated by GSH depletion from the negative regulation of the Nrf2-ARE pathway. Lipids and proteins from cells are targets of ROS, producing structural damages in membranes and leading to major REDOX imbalance [55,56,57]. Our results indicated that IGF-II protects the neurons against ROS production and the dramatic increase in the levels of the cell damage markers AOPP and LOOH; the reduction in TAS and GPX activity is also counteracted. These changes agree with previous results in cellular models of PD [24,27] and after administration of CORT in neuronal cultures [12], where IGF-II prevented oxidative balance and GPX expression.

As with other neurodegenerative diseases, PD is strongly associated with the balance in free radical formation/scavenging processes [57,58], where mitochondria are some of the main sources of ROS production and energy [3,59,60,61]. In this combined model of drug-induced PD and emotional stress, as in other mitochondrial toxic conditions in neuronal cultures [12,13,17,35,41], we found decreases in mitochondrial function and increases in ROS production inducing neuronal damage. With the inclusion of IGF-II in the treatment, we observed a preservation of mΔΨ and ROS balance and a partial protection of the cellular bioenergetic parameters: basal O_2_ consumption, maximal and ATP-linked respirations, and the bioenergetic reserve, also known as spare respiratory capacity (SRC). The use of SRC by neurons ranges from 7% (resting situations) up to 80% (firing neurons) [62]. Thus, its use helps neurons meet increased energy demands, which is crucial to maintaining cell homeostasis against different types of cellular stress [62], as its deterioration is fatal to neurons. IGF-II leads to SCR maintenance, avoiding cell death. We therefore hypothesised that both activation of antioxidant systems and the increase in mitochondrial function contributed to the cytoprotective effects of IGF-II against oxidative distress. Thus, IGF-II improves mitochondrial function in skeletal muscle and liver under stress conditions [63,64]. In the combined model used in this study, the administration of both drugs inhibited ATP-linked respiration. However, individual CORT administration maintains initial and pressure ATP turnover [17], which could denote an adaptive priming to supply the energy needed for the accurate neuronal functioning under stress conditions [49], leading to an increase in mitochondrial activity with a consequent ROS production and, lastly, a decrease in mitochondrial activity and dysfunction over time. As an anabolic molecule, the role of IGF-II in increasing ATP synthesis is well known. Thus, the effect observed could be related to increased ATP availability as was demonstrated previously [26]. Concerning proton leak, the co-treatment with IGF-II and both drugs prevented its increase, improving mitochondrial efficiency [65], probably through the preservation of membrane oxidation and/or uncoupling protein modulation. The antioxidant functions of mild uncouplers, i.e., compounds that uncouple the processes of phosphorylation and mitochondrial electron transport and that ultimately maintain the ATP at an adequate level to meet cell metabolic demands, have led to an explosion of interest in the use of these molecules in the treatment of several pathologies, including neurological disorders at present [66]. Our previous study did not suggest the role of IGF-II as a mild uncoupler or a conventional antioxidant molecule, because the effects of IGF-II depend on its interaction with IGF-IIR [24]. However, IGF-II could modulate possible mild uncouplers [66]; specific studies are needed to confirm this hypothesis. Similarly, to compare IGF-II neuroprotection to the role of conventional antioxidants, experiments indicating whether IGF-II can act as a conventional antioxidant should be carried out.

PKC’s role in the regulation of neuronal mitochondrial metabolism is known [67], which could lead to protective effects. Thus, in the organotypic hippocampal culture model of neurodegeneration, PKC inhibition enhanced cell death after excitotoxic damage and could be acting under the influence of postischemic ROS and ATP synthesis [68]. Moreover, PKC activation could lead to Nrf2 transcriptional activation and the maintenance of cardiomyocyte antioxidant defences in post-conditioned rat hearts [69], suggesting an essential role of PKC/Nrf2 signalling activation (see below). In our model, the combination of both drugs decreased pPCKα/β expression, whereas with the inclusion of IGF-II in the treatment, we observed a preservation of its expression, suggesting that the neuroprotective effect of IGF-II could involve PKC activation. This IGF-II-related increase in PKC phosphorylation was previously found in cardiomyoblast cells [70]. Further studies of the specific contribution of this PKC-mediated mechanism are required. Therefore, the beneficial effect of IGF-II on mΔΨ and cellular bioenergetic parameters could be mediated by PKC activation and would protect energy resources in PD with mild to moderate stress, contributing to maintaining the neuron integrity and its functions and making cells less vulnerable to neurodegeneration, as shown in Figure 5. The increase in FJ staining after incubation with CORT and MPP^+^ indicated high levels of neurodegeneration. This result was in agreement with those observed in animal PD models, where mild stress is an environmental risk factor that leads to progressive neurodegeneration [50]. In our study, the FJ dye increase produced with the incubation of both drugs was partially counteracted by co-incubation with IGF-II, leading to a protective effect that was also found in a cellular PD model and a cellular oxidative stress model induced by CORT [12,24]. Since accumulation of ROS and mitochondrial dysfunction are two of the main mechanisms eliciting neurodegeneration [71], the IGF-II effect could be related to improvement of mitochondrial function, as mentioned above, but also to a decrease in ROS production. Combined models of PD and mild to moderate stress have also examined the relatively progressive dopaminergic neuron loss and nigral cell degeneration. Thus, they exposed that emotional stress produced a worsening of the PD symptoms, accelerating dopaminergic cell degeneration in the substantia nigra [49,72]. In human studies, this possible relationship has also been suggested [7]. One of the hallmarks of PD is the decrease in dopamine biosynthesis [73], TH being the rate-limiting enzyme and therefore a marker for dopamine neurons. We evaluated the effect of IGF-II on the toxicity induced by CORT and MPP^+^ treatment in TH levels, and we found that IGF-II maintained the TH expression, which agreed with our previous results in drug-induced cellular PD [24].

The transcription factor Nrf2 is considered a REDOX-sensitive factor involved in oxidative damage adaptation and inflammation by its role in the regulation of around 200 cytoprotective proteins [74,75,76]. Nuclear factor (erythroid-derived 2)-like 2 enhances the transcription of antioxidant response elements, leading to the emergence of Nrf2 as a key target for a number of disorders including neurodegenerative diseases. Thus, Nrf2 is believed to suppress the harmful oxidative distress and related neuroinflammation in PD [77]. In this study, incubation with the two drugs indicated a decrease in Nrf2 translocation to the nucleus in agreement with previous results found in different neuronal oxidative cell models [24,35], which could contribute to the GPX decrease observed. The decrease in Nrf2 translocation observed in the combined model of PD and mild to moderate stress could be attributed to a decrease in Nrf2 expression [24,78,79], to Nrf2 metabolism alterations [80], or to a reduction in translocation by itself [81,82], induced by MPP^+^/MPTP; but it could also be related to the CORT effect mediated by the interaction between GR and Nrf2, owing to GR signalling blockage of Nrf2-mediated cytoprotection from oxidative distress [83]. In any case, IGF-II co-incubation prevented the Nrf2 nuclear translocation as a direct IGF-II consequence and/or trough interaction with other factors, inhibiting the GR translocation to the nucleus (see below and Figure 7).

Stress has long been hypothesised to contribute to PD neuropathology, possibly by its deleterious effects on midbrain neurons [14]. The role of glucocorticoids acting through their interaction with GR in the pathophysiology progression of PD was observed. Indeed, basal plasmatic cortisol levels are increased in PD patients, suggesting dysregulation in the HPA axis, which affects GR activity [15]. In an animal model of drug-induced PD, the chronic administration of CORT increased GR density in motor areas [45]. Accordingly, GR density is believed to be involved in PD neurodegenerative regulation [18], which may exacerbate symptoms and neuronal loss. As shown in Figure 1a, co-incubation with MIFE as a GR antagonism in the treatment media produced a 2-fold decrease in cell death compared to the administration of both drugs. Regarding TH expression, the administration of MIFE partially abolished the toxic effect, confirming the main effect of the stressor on TH toxicity aggravation in the model. Moreover, in our study, the decreases in pPKCα/β expression and nuclear Nrf2 translocation caused by the drugs were counteracted when MIFE was administered. Finally, the nuclear GR expression increased after CORT and MPP^+^ administration, whereas the co-incubation of both drugs with MIFE showed an antagonist effect on CORT-induced GR translocation into the nucleus. Again, co-treatment with IGF-II and both drugs prevented the nuclear translocation. These results in Nrf2 and GR nuclear translocation suggested that in this combined model of PD and mild to moderate stress, where CORT plays a central role in the synergistic toxic effect observed, IGF-II could be mainly acting as a PKC and Nrf2 activator mediated by GR signalling pathways. Although the correlations found supported our model, future studies to confirm these hypotheses and explore the complete molecular mechanisms of IGF-II action are needed.

All these findings revealed that, in the cellular model used in this study based on CORT, an endocrine response marker to stress, and the dopaminergic neurotoxin MPP^+^, there was an increase in neurodegeneration and cell death as a consequence of an increase in oxidative distress and mitochondrial dysfunction. The molecule IGF-II showed neuroprotective activity against the toxic synergistic effect of both drugs by promoting pPKCα/β expression and Nrf2 antioxidant response in a GR-dependent pathway and preventing oxidative cell damage and maintaining mitochondrial function.

## 5. Conclusions

This work revealed the potential neuroprotective role of the hormone IGF-II in a cell model of PD aggravated by mild to moderate hormonal stress. The capacity of IGF-II to protect nigral dopamine neurons against the mitochondrial-oxidative damage induced by CORT and MPP^+^ was demonstrated. Considering these potential beneficial effects of IGF-II, the molecule is proposed as a potential therapeutic tool for treating and preventing disease progression of PD patients suffering mild to moderate emotional stress.

## Data Availability

Data are contained within the article.

## Supplementary Materials

The following supporting information can be downloaded at: https://www.mdpi.com/article/10.3390/antiox11010041/s1. List of abbreviations; Figure S1: Representative immunocytochemistry stain for GR and Nrf2.
