# Supplementary material for: Insulin-like Growth Factor II Prevents MPP+ and Glucocorticoid Mitochondrial-Oxidative and Neuronal Damage in Dopaminergic Neurons
Source: Antioxidants (Basel). 2021 Dec 24;11(1):41. doi: 10.3390/antiox11010041 (PMC8773450; doi:10.3390/antiox11010041)
Supplement: Supplementary file 1 [file antioxidants-11-00041-s001.zip › antioxidants-1486437-supplementary.pdf]

Supplementary Material

1. List of Abbreviations

(AOPP) Advanced-oxidation protein products
(CO) Control cells
(CORT) Corticosterone
(FCCP) Carbonyl cyanide-4-(trifluoromethoxy)phenylhydrazone
(FJ) Fluoro-Jade B™
(GC) Glucocorticoid
(GPX) Glutathione peroxidase
(GR) Glucocorticoid receptors
(HPA) Hypothalamic–pituitary–adrenal axis
(IGF-IR) Insuline-like growth factor I receptor
(IGF-II) Insulin-like growth factor II
(IGF-IIR) IGFII/Mannose 6-Phosphate receptor
(InRs) Insulin receptors
(JC-1) 5,5,6′,6′-tetrachloro-1,1′,3,3′-tetraethyl benzimidazolcarbocyanine iodide
(LDH) Lactate dehydrogenase
(LOOH) Lipid hydroperoxides
(MIFE) Mifepristone
(MPP+) 1-methyl-4-phenylpyridinium
(m$\Delta\Psi$) Mitochondrial membrane potential
(Nrf2) Nuclear factor (erythroid-derived 2)-like 2
(OCR) Mitochondrial oxygen consumption rate
(PD) Parkinson's disease
(PKCα) Protein kinase C alpha
(ROS) Reactive oxygen species
(SRC) Spare respiratory capacity
(TAS) Total antioxidant status
(TH) Tyrosine hydroxylase

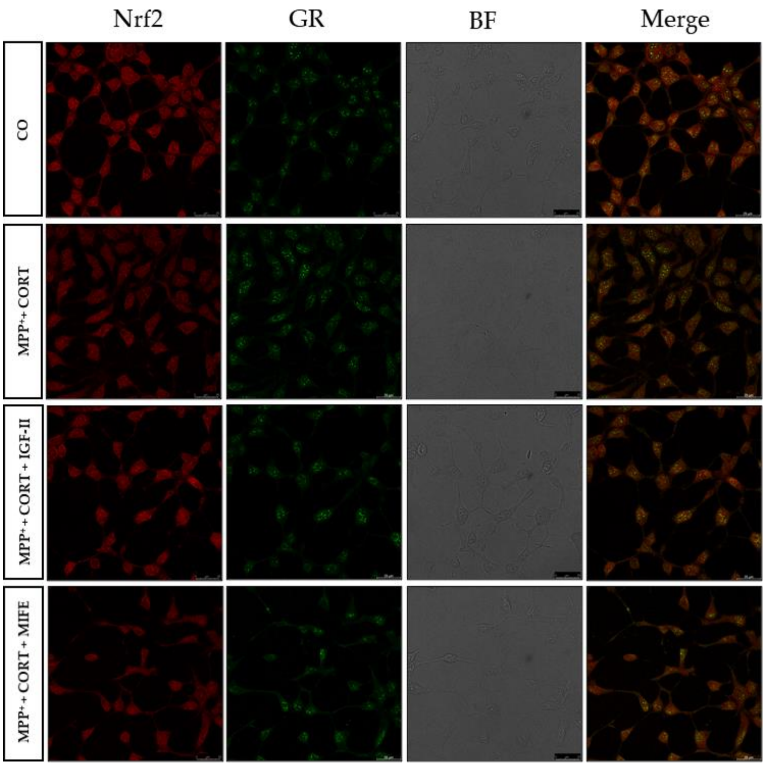

Figure S1. Study of intracellular signalling pathways in SN4741 neuronal cells after 2.5 h of incubation with the combination of corticosterone and MPP^+ (CORT + MPP^+) in the presence or absence of IGF-II. Representative immunocytochemistry stain for GR and Nrf2 (63X). CO: control cells. BF: Bright Field.
